# Evaluation of tumour surveillance protocols and outcomes in von Hippel-Lindau disease in a national health service

**DOI:** 10.1038/s41416-022-01724-7

**Published:** 2022-02-19

**Authors:** Eamonn R. Maher, Julian Adlard, Julian Barwell, Angela F. Brady, Paul Brennan, Jackie Cook, Gillian S. Crawford, Tabib Dabir, Rosemarie Davidson, Rebecca Dyer, Rachel Harrison, Claire Forde, Dorothy Halliday, Helen Hanson, Eleanor Hay, Jenny Higgs, Mari Jones, Fiona Lalloo, Zosia Miedzybrodzka, Kai Ren Ong, Frauke Pelz, Deborah Ruddy, Katie Snape, James Whitworth, Richard N. Sandford

**Affiliations:** 1 Department of Medical Genetics, University of Cambridge and Cambridge University Hospitals NHS Foundation Trust, Cambridge, CB2 0QQ UK; 2 Clinical Genetics Department, Chapel Allerton Hospital, Leeds, LS7 4SA UK; 3 Department of Clinical Genetics, University Hospitals of Leicester, Leicester, LE1 5WW UK; 4 North West Thames Regional Genetics Service, London North West University Healthcare NHS Trust, Northwick Park Hospital, Leicester, HA1 3UJ UK; 5 Northern Genetics Service, Newcastle Hospitals NHS Foundation Trust, Newcastle, UK; 6 Department of Clinical Genetics, Sheffield Children’s Hospital NHS Foundation Trust, Sheffield, S10 2TH UK; 7 Wessex Clinical Genetics Service, Level G, Princess Anne Hospital, Coxford Road, Southampton, SO16 5YA UK; 8 Northern Ireland Regional Genetics Service, Belfast City Hospital, Belfast, BT9 7AB UK; 9 Department of Clinical Genetics, Queen Elizabeth University Hospital, Glasgow, G51 4TF UK; 10 South East of Scotland Clinical Genetics, Western General Hospital, Edinburgh, EH4 2XU UK; 11 Clinical Genetics Service, Nottingham University Hospitals NHS Trust, Nottingham, NH5 1PB UK; 12 Manchester Centre for Genomic Medicine, St Mary’s Hospital, Manchester University Hospitals NHS Foundation Trust, Manchester, UK; 13 Oxford Centre for Genomic Medicine, Oxford University Hospitals NHS Foundation Trust, Oxford, OX3 7HE UK; 14 Department of Clinical Genetics, St George’s University Hospitals NHS Foundation Trust, London, UK; 15 Department of Clinical Genetics, Great Ormond Street Hospital, London, WC1N 3JH UK; 16 Liverpool Centre for Genomic Medicine, Liverpool Women’s Hospital, Liverpool, L8 7SS UK; 17 Medical Genetics Group, University of Aberdeen, Aberdeen, UK; 18 Clinical Genetics Department, Birmingham Women’s and Children’s NHS Trust, Birmingham, B15 2TG UK; 19 All Wales Medical Genomics Service, Cardiff and Vale University Health Board, Cardiff, CF14 4XW UK; 20 Department of Clinical Genetics, Guys and St Thomas’ Hospital, London, SE1 9RTX UK

**Keywords:** Translational research, Risk factors, Urological cancer

## Abstract

**Background:**

Von Hippel-Lindau (VHL) disease is an inherited tumour predisposition syndrome and a paradigm for the importance of early diagnosis and surveillance. However, there is limited information on the “real world” management of VHL disease.

**Methods:**

A national audit of VHL disease in the United Kingdom.

**Results:**

VHL disease was managed mostly via specialist clinics coordinated through regional clinical genetics services (but frequently involving additional specialties). Over the study period, 19 genetic centres saw 842 individuals (393 males, 449 females) with a clinical and/or molecular diagnosis of VHL disease and 74 individuals (35 male, 39 female) with a prior risk of 50% (affected parent). All centres offered retinal, central nervous system and abdominal surveillance to affected individuals and at-risk relatives though surveillance details differed between centres (but complied with international recommendations). Renal lesions detected on the first surveillance scan were, on average, larger than those detected during subsequent scans and the larger the diameter at detection the greater the likelihood of early intervention.

**Conclusions:**

In a state-funded health care system individuals with a rare inherited cancer predisposition syndrome are generally able to access appropriate surveillance and patient management is improved compared to historical data. The “real world” data from this study will inform the future development of VHL management protocols.

## Introduction

Von Hippel-Lindau disease is a rare autosomal dominantly inherited disorder characterised by predisposition to multiple tumour types and caused by germline pathogenic variants in the *VHL* tumour suppressor gene [1–4]. The expression of VHL disease is quite variable, onset may be in infancy or in the seventh decade [5, 6]. The major tumour types are retinal and central nervous system (CNS) haemangioblastomas, renal cell carcinoma (RCC), phaeochromocytoma, pancreatic neuroendocrine tumours (PNET) and endolymphatic sac tumours (ELST). In addition, renal, pancreatic and epididiymal cysts are common with increasing age [3, 5, 6]. Clinical diagnostic criteria for VHL disease were defined in 1964 (1) a single VHL-related neoplasm such as a haemangioblastoma or RCC if there is a confirmed family history of VHL disease or, (2) in sporadic cases, two haemangioblastomas or a haemangioblastoma and a second VHL-related tumour) [7]. During the 1980s and 1990s there were multiple descriptions of large kindreds and multicentre patient cohorts that established the high age-related risks of retinal and CNS haemangioblastomas and RCC (each >70% lifetime risk) in most families, and interfamilial variations in frequency of phaeochromocytoma susceptibility [5, 8–11]. Based on these high age-related risks it was suggested that affected individuals and their at-risk relatives should be offered regular surveillance to detect complications (especially retinal haemangioblastomas and RCC) at an early stage in order to reduce morbidity and mortality [5, 12]. Subsequently it was reported that the adoption of surveillance programmes was associated with an increase in the proportion of retinal haemangioblastoma that were detected at an early (asymptomatic) stage, a reduction in the age at diagnosis of RCC and increased survival [13, 14]. With the adoption of annual renal surveillance, it was recognised that RCC could be detected at an early stage and monitored until they reached a diameter of 3 cm before surgical treatment, with minimal risk of metastatic disease [15, 16]. Furthermore as there is a high risk of further ipsilateral and contralateral RCC, the recommended management is nephron-sparing subtotal nephrectomy (or localised ablation using RFA or cryosurgery) in order to preserve renal function for as long as possible [16, 17].

The identification of the *VHL* tumour suppressor gene in 1993 and the subsequent characterisation of >1000 germline pathogenic variants has enabled genetic diagnosis and pre-symptomatic testing to be made available to most affected families and facilitates early diagnosis, particularly in those without a family history (up to 20% of probands result from de novo mutations) [1, 18, 19]. Pre-symptomatic testing is offered from early childhood and there is a high uptake, with those relatives who test negative being released from annual surveillance programmes and so increasing the cost-effectiveness of surveillance [20]. Over the past two decades there has been a general consensus among expert opinions of the value of surveillance to detect VHL-related complications early (particularly for ocular and renal tumours) though the details (e.g. age at which screening is commenced and imaging modalities) can vary between centres and countries [2, 4, 21, 22]. However, there is little information on the “real world” implementation of such surveillance programmes within large countries and the extent of inter-centre variability (and its consequences). To address this knowledge deficit an audit was initiated to define the characteristics of services for patients and families with VHL disease across the United Kingdom (population ~65,110,000 Office for National Statistics 2016). Here we report the findings of this survey.

## Methods

### Audit details

Twenty-two regional genetics centres were contacted and asked to complete a standardised questionnaire (see Supplementary material) to collect data on relevant individuals who have been under follow-up for all or part of the period 2012–2017. Individuals were included in the audit if (a) they had a clinical and/or molecular diagnosis of VHL disease or (b) they were at 50% risk of inheriting VHL disease and had not undergone predictive genetic testing. A clinical diagnosis of VHL is defined according to the criteria specified in Genereviews: https://www.ncbi.nlm.nih.gov/books/NBK1463/): 1. A simplex case (i.e. an individual with no known family history of VHL syndrome) presenting with two or more characteristic lesions (two or more haemangioblastomas of the retina, spine, or brain or a single haemangioblastoma in association with a visceral manifestation (e.g. renal cell carcinoma, adrenal or extra-adrenal phaeochromocytoma, endolymphatic sac tumour (ELST), or neuroendocrine tumour of the pancreas (pNET)) and 2. an individual with a positive family history of VHL disease in whom one or more of the following syndrome manifestations was present: retinal haemangioblastoma, spinal or cerebellar haemangioblastoma, adrenal or extra-adrenal pheochromocytoma, renal cell carcinoma, multiple renal and pancreatic cysts). Patient identifiable data was not collected.

The audit was carried out against screening guidelines published Maher et al. [2]. These screening guidelines were selected because (a) they had been published and widely read several years before the study period and (b) they were from a European group and would more closely reflect medical practice in the UK than recommendations from USA groups.

#### Screen for retinal angioma

Annual ophthalmic examinations (direct and indirect ophthalmoscopy), beginning in infancy or early childhood.

#### Screen for CNS haemangioblastoma

MRI scans of the head for every 12–36 months, beginning in adolescence.

#### Screen for renal cell carcinoma and pancreatic tumours

MRI (or ultrasound) examinations of the abdomen every 12 months, beginning from the age of 16 years.

#### Screen for phaeochromocytoma

Annual blood pressure monitoring and 24-h urine studies for catecholamine metabolites. More intense surveillance (e.g. annual measurement of plasma normetanephrine levels, adrenal imaging, beginning from the age of 8 years should be considered in families at high-risk for phaeochromocytoma).

### Statistical analysis

*t*-testing and *χ*^2^ analysis were performed as appropriate. Statistical significance was designated as 5%.

## Results

### Organisation of NHS specialist services for VHL disease

Twenty-two Regional Genetics Centres were contacted to participate in the national audit and completed the audit questionnaire (see Supplementary material). Four centres reported that they did not have a formal VHL surveillance clinic (one because of low numbers, one because there were two clinics running at other hospitals in their region and two undertook individual non-specialist clinic reviews). Of the 18 formal surveillance clinics for which information was obtained, 16 were led by the clinical genetics service and two by the endocrinology service. Of the clinical genetics service led clinics, most (11/16) were multidisciplinary. The most frequent specialties represented in the multidisciplinary clinics were ophthalmology (*n* = 8), endocrinology (*n* = 4), urology (*n* = 3) followed by paediatrics, neurology, nephrology, radiology (each *n* = 1).

### Characteristics of individuals attending NHS specialist services for VHL disease

Data were obtained from 22 UK regional genetics centres. Overall 842 individuals (393 males, 449 females) with a clinical and/or molecular diagnosis of VHL disease attended one of the centres over the audit period (2012–2017). A pathogenic VHL variant had been detected in 750 individuals but not in 64 (diagnostic rate 92%). In addition, 74 individuals (35 male, 39 female) with a prior risk of 50% (affected parent) were also seen. Individuals at 50% risk were screened as per individuals with confirmed VHL disease in all centres until (a) their status was resolved by genetic testing or (b) they reached the age at which screening was discontinued if no evidence of VHL disease had appeared. The age at which screening was discontinued varied between 40 and 65 years with a median age of 50 years.

The number of individuals seen at each centre ranged from 1 to 101 (mean 38.2, median = 33). The UK population in 2016 was 65.11 million (www.ons.gov.uk) giving a maximum prevalence of 1.29 per 100,000 population (1 per 77,340) but excluding the patients from the two regions where data ascertainment appeared to be incomplete gave an estimated maximum prevalence of 1.4 per 100,000 population (1 per 71,480). Counting half of the individuals at 50% prior risk further increased this to 1.46 per 100,000 population (1 per 68,493).

There were 33 deaths over the study period (mean age at death 50.4 years, range 25–82 years) and 17 deaths were considered to be related to a complication of, or treatment for, VHL disease (mean age at death 48.3 years, range 25 to 68 years). For 11 cases, mean age at death 58.8 years, range 40 to 82 years, the cause of death was not considered to be linked to VHL disease (e.g. myocardial infaction) and for 5 cases the relationship to VHL disease was uncertain (mean age at death 47.8 years, range 31 to 57 years).

### Surveillance programmes in clinical genetics centres offering VHL disease surveillance

#### Retinal haemangioblastoma

Using the standard recommendation of *“annual ophthalmic examinations (direct and indirect ophthalmoscopy), beginning in infancy or early childhood”*, the majority (15/18) centres met the audit standard with most centres (13/18) commenced retinal surveillance at age 5 years (two centres started earlier (age 2 or 3 years)) but three centres started later (e.g. 5–8 years, 5–10 years or 7–10 years).

#### Central nervous system haemangioblastoma

The surveillance recommendation that responses were assessed against was “MRI scans of the head for every 12–36 months, beginning in adolescence”. All centres offered some surveillance for cerebellar and spinal haemangioblastomas, though there were inter-centre variations according to whether regular surveillance was performed (for brain in 16 centres for spinal cord in 13 centres) or just a baseline MRI (for brain and spinal cord in 5 centres at median age 16 years (range 11–18 years). The first CNS MRI scan was generally performed 14 to 16 years (58% of centres, range 8–18 years). For those centres that performed regular MRI scans the most common interval was 36 months for both brain and spine (88% and 92% of centres).

#### Abdominal imaging

To detect renal cell carcinoma and pancreatic tumours the selected surveillance protocol recommended “MRI (or ultrasound) examinations of the abdomen every 12 months, beginning from the age of 16 years” [2]. Only one genetics centre did not commence abdominal imaging at age 16 years (MRI was offered from 18 years) but 12 centres offered abdominal imaging from an earlier age (youngest age 8 years). The imaging modality varied between centres e.g. MRI only (*n* = 9), ultrasonography only (with CT/MRI for suspected lesions) (*n* = 6) or combinations (e.g. alternating) of ultrasound and MRI (*n* = 6).

#### Phaeochromocytoma and paraganglioma (PPGL) surveillance

All centres offered biochemical testing (urine or plasma catecholamine measurements) usually commencing between 5 and 10 years of age (age 11 in one centre). Several centres commented that they commenced surveillance earlier for “higher risk” cases (e.g. family history of PPGL and/or known PPGL-associated VHL variant).

### Renal cell carcinomas in VHL disease

**Fig. 1:**
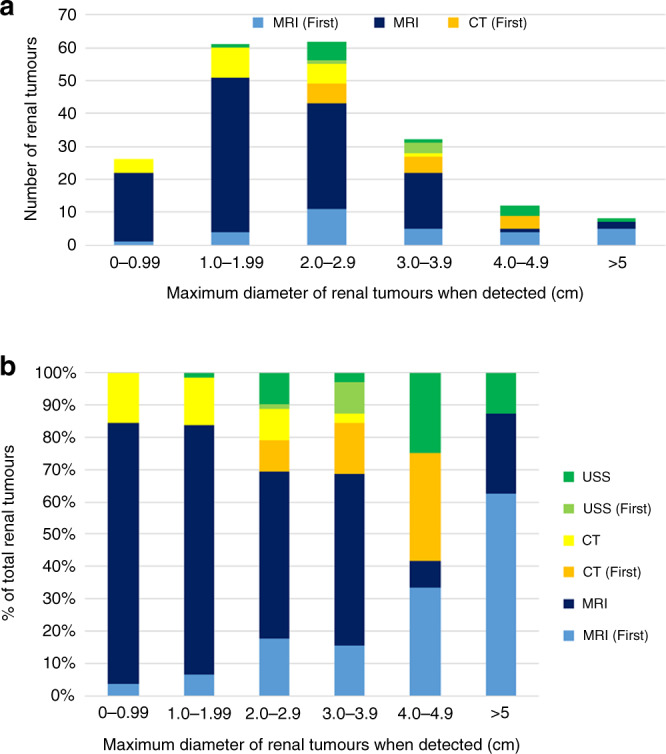
Maximum diameter of renal tumours when detected according to imaging modality and whether it was the first scan of that type. **a** Distribution of renal tumour sizes and imaging: MRI (First) = tumours detected on first renal MRI scan in that individual; MRI = tumours detected on a MRI scan in individuals with previous MRI or CT scans; CT (First) = tumours detected on first renal CT scan in that individual; CT = tumours detected on a CT scan in individuals with previous MRI or CT scans; USS (First) = tumours detected on first renal ultrasound scan in that individual; USS = tumours detected on a renal ultrasound scan in individual with previous renal ultrasonography. **b** Details of imaging modality to detect tumours of different sizes (same data as in (**a**) but expressed as a % of total tumours of that size).

Over the study period, 242 RCC were detected in 170 individuals (35 with multiple lesions (median 2, maximum 7 tumours)). The mean age at detection of a RCC was 39.4 years (SD ± 12.7) and the mean maximum diameter of the lesion when detected was 2.16 ± 1.24 cm. 53 lesions in 31 patients were detected on the first renal imaging (mean maximum diameter 3.07 ± 1.28. This mean maximum diameter of lesions detected on the first renal scan was significantly larger than those that were detected in subsequent scans (mean maximum diameter of 1.88 ± 1.11 cm (*t* = 6.34*, P* < 0.0001)). The most frequent imaging modality for detecting a RCC was MRI (*n* = 181 lesion) followed computed tomography (CT) (*n* = 40) and ultrasonography (*n* = 18) (3 not specified). The size distribution (maximum diameter) of the renal lesions detected subdivided by modality and whether first scan or not is shown in Fig. 1.

**Fig. 2:**
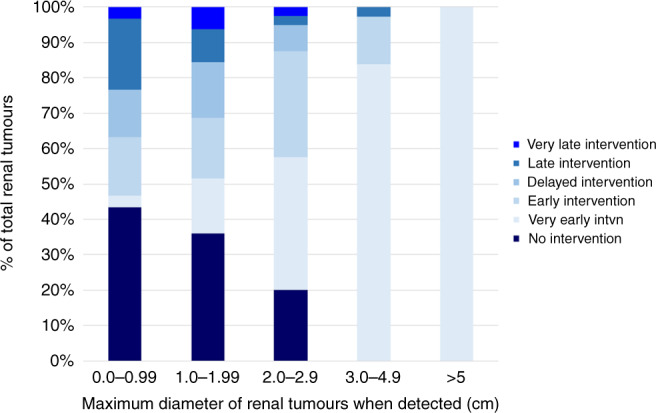
Outcome over the study period of detected RCC according to tumour size (maximum diameter in cm). No intervention = tumour remained under surveillance; very early intvn= proceeded to intervention (surgical removal or ablative procedure) within 12 months; early intervention = proceeded to intervention within 12–35 months of detection; delayed intervention = proceeded to intervention between ≥3 and <5 years of detection; late intervention = proceeded to intervention between ≥5 and <10 years of detection; very late intervention = proceeded to intervention ≥10 years after detection.

The treatment outcome was available for 229 RCC: 51 were under observation, 134 were treated by surgical excision and 44 by ablation (radiofrequency ablation or cryoablation). For those that proceeded to surgery or ablative therapy, there was a wide variability in the timescale to intervention with 32.3% being treated within 12 months of detection and 3% more than 10 years later. The relationship between maximum diameter when detected and outcome (intervention or no intervention) is illustrated in Fig. 2. This demonstrates that, as the maximum tumour diameter increases, the number of lesions with no intervention falls and there is a trend from late to early intervention (some interventions for small RCC were probably performed when other larger tumours in the same kidney were being treated) (chi-squared testing for trend of no/very late/late/delayed intervention versus early/very early intervention with increasing tumour diameter *χ*^2^ = 59.4, *P* < 0.0001).

Fifteen individuals (1.8%) developed end-stage renal failure during the study period at a mean age of 40.4 years (range 22–61 years) and 7 of these went on to have a renal transplant (at mean 37.3 years, range 29–53 years).

### Central nervous system haemangioblastomas in VHL disease

**Fig. 3:**
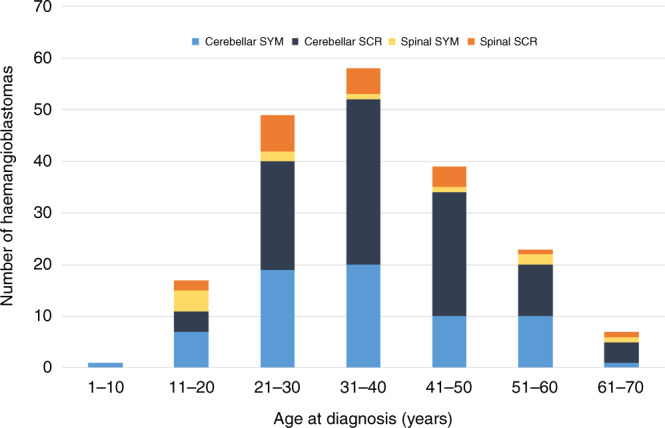
Age distribution of haemangioblastomas. Age distribution of cerebellar and spinal haemangioblastomas according to whether they were detected through routine surveillance (cerebellar SCR and spinal SCR) or because of symptoms (cerebellar SYM or Spinal SYM).

Over the study period, 217 haemangioblastomas were detected in 183 individuals (25 with multiple haemangioblastomas (median 2, maximum 3 tumours) at a mean age of 37.04 years (range 9–66 years). 38% of haemangioblastomas were detected following symptoms and 62% were asymptomatic and diagnosed on routine surveillance imaging. Location of the haemangioblastoma was specified for 215: 176 (82%) of haemangioblastomas were located in the cerebellum, 35 (16%) in the spine and 4 (2%) in the brain stem. The age distribution of the haemangioblastomas diagnosed in the study period (subdivided by location and symptomatic status) is shown in Fig. 3.

#### Pancreatic neuroendocrine tumours (PNETs) in VHL disease

A PNET was present in 36 patients (mean age 38.5 ± 13.2 years) in the study period (30 with a single tumour and 6 with multiple PNETs). The earliest recorded age at diagnosis was 18 years. The mean maximum diameter at diagnosis was 2.05 ± 1.28 cm; range 0.9–6.3 cm). 27 PNETs were detected by MRI and 6 and 3 by computer tomography and ultrasonography respectively. In 9 cases, the PNET was detected on the first MRI or CT scan performed (mean maximum diameter 2.01 ± 0.96 cm. Nineteen patients were under surveillance, 15 had undergone surgical intervention and 3 were being managed medically e.g. lanreotide). Comparing centres that performed MRI scans at least once every two years for abdominal surveillance and those that performed less frequent MRI scans (or ultrasound only), there was a significantly increased frequency of PNET detected in the former ((6.1 and 2.6 per 100 patients respectively, *p* = 0.017).

#### Endolymphatic sac tumours

were diagnosed in 11 patients (1.3% of the cohort) during the study period. In 3 patients the diagnosis was made in an asymptomatic individual through screening investigation and in 8 cases the patients were symptomatic at diagnosis.

### Very early age at diagnosis of VHL-related neoplasia

Surveillance programmes look to balance comprehensive early detection with cost efficiency. To identify the extent to which current surveillance recommendations might miss some unusually early presentations of VHL-related tumours, each of the centres was asked to specify the number of such cases they had seen over the study period. There was one case of a retinal haemangioblastoma and none of a CNS haemangioblastoma younger than age 5 years, two individuals with RCC were diagnosed before 18 years and 7 with phaeochromocytoma were diagnosed <10 years (youngest age 7 years).

## Discussion

We report the results of a national audit of VHL disease performed across 22 regional clinical genetics centres. The number of patients seen in each centre was variable with higher numbers in centres with a larger catchment area and/or longstanding dedicated specialist clinics. Within the UK there is no nationally commissioned service for VHL disease surveillance, but there was a high degree of similarity between the different regional services. In most centres the specialist clinic was led by staff from clinical genetics. This arrangement reflects the early establishment of dedicated VHL specialist clinics in some UK genetics centres [5, 12, 23] and the multisystem nature of VHL disease that means that it does not entirely fall under any specific medical or surgical specialty. We found variability in the organisation of VHL clinical services with some centres having up to four different specialties represented in their clinic, whilst other centres offered a less centralised clinic model. A multidisciplinary or “one-stop” service model can be more convenient for patients and families and reduce the number of hospital visits, though it is unknown whether such an arrangement improves clinical outcomes. With a large number of clinical specialties potentially involved in clinical care, our experience is that a key factor for an optimal service is that a named clinician coordinates their multifaceted care. This audit was completed before the COVID-19 pandemic, during which some centres adopted telemedicine approaches to clinical care. We anticipate that some of the developments put in place during the pandemic (e.g. virtual clinics and digital retinal imaging techniques [24, 25]) might be retained in order to improve efficiency and patient compliance.

VHL disease provides an exemplar of an inherited cancer syndrome for which surveillance programmes have reduced morbidity and mortality through earlier diagnosis [13, 14, 26]. However, worldwide there is little information available on access to appropriate surveillance outside of centres with a research interest in VHL disease. We assessed the screening protocols for all the centres against that specified in a review written by an international (European) group of experts [2]. The majority of centres audited met the selected audit standard for surveillance, but in a few centres retinal and abdominal imaging commenced later than was recommended, and not all centres offered regular ongoing brain and/or spinal cord imaging (as specified in the recommended protocol). The published surveillance programme that we selected for assessing current care made broad recommendations that would be applicable to a range of health care systems (e.g. that all patients should be offered annual abdominal imaging but the modality of imaging was not defined) [2]. It is generally agreed that MRI is superior to ultrasonography in detecting small renal masses (though the latter is operator dependent) and that annual CT scanning should be avoided because of radiation load [2, 22, 27]. We found that most, but not all centres, were offering MRI alone or alternating with ultrasonography, rather than ultrasonography alone for abdominal surveillance. Though it was not possible to make a direct comparison of MRI and USS for detecting small renal tumours, we note that our data suggested that renal masses are detected earlier when MRI is used for surveillance (particularly when lesions are not detected on the first scan) (see Fig. 1a, b)). Furthermore, we found that the smaller the renal mass at detection the less likely very early or early intervention (within 12 months and within 12–36 months respectively) would be required (Fig. 2). We note a recent recommendation that the frequency of MRI abdominal surveillance might be reduced to biannually [22] but, in the “real world” series reported here, there were instances of renal lesions being diagnosed by MRI at ≥3 cm (despite this not being the first scan). The format of the audit does not allow us to say what the reasons for this might be (e.g. it could be that the interval between scans was longer than the intended through compliance or organisational issues) but this finding suggests that any move to lengthen screening intervals at a national level would need to be carefully monitored as, outside of well-resourced specialist centres, such a change could increase the risk of delayed diagnosis of RCC and potentially also for PNETs.

It was apparent that each centre appeared to be following the “3 cm rule” i.e. observing small RCC until they reach this size and then undertaking renal sparing surgery (though when ablative strategies were used treatment might be instigated for lesions 2–3 cm diameter) [15–17]. We note that though the frequency of early intervention increased with tumour size, there was apparently considerable heterogeneity in progression rates with some RCC < 2 cm diameter proceeding to intervention within 12 months. Again, this finding would suggest that if screening intervals are to be lengthened, then in “real-world scenarios” careful monitoring of any lesions detected would be initially required to establish growth rates of individual lesions. Over the past three decades, the adoption of renal surveillance programmes and nephron-sparing surgery in VHL disease has reduced the risk of death from RCC and the incidence of end-stage renal failure following renal surgery [5, 12, 23].

Most variability between the recommended surveillance and that which was being offered in practice was for central nervous system haemangioblastomas, with multiple centres not offering regular imaging. Nevertheless, it was noticeable that 62% of cerebellar and spinal haemangioblastomas diagnosed in the study period were detected at an asymptomatic stage by a surveillance MRI scan. This represents a major shift over the past three decades in UK clinical practice, as in earlier studies, almost all haemangioblastomas presented symptomatically [5]. Though, in general, completely asymptomatic haemangioblastomas are surgically removed only infrequently and non-cystic haemangioblastomas may not enlarge over a period of years (and so the prognosis of small asymptomatic lesions can be difficult to predict), CNS imaging can detect asymptomatic syringomyelia which may require intervention and also identify markers of progression such as rapid growth and an associated cyst [28–30]. Knowledge of asymptomatic lesions that are progressively enlarging may allow more considered planning of surgical intervention and reduce the risk of late presentation. The availability of medical treatments such as the HIF-2 antagonist belzutifan for the treatment of VHL disease will provide further impetus for early detection [31].

The age at which surveillance should commence for individual manifestations of VHL disease can be contentious. Rare cases of exceptional early onset can bring pressure to start surveillance sooner. However, whilst the results of this audit confirm that some manifestations of VHL disease can present before the age at which surveillance is commenced, in most centres such occurrences are rare, with just one case of a retinal haemangioblastoma diagnosed younger than age 5 years, and 7 children with phaeochromocytoma diagnosed before age 10 years. Some surveillance protocols recommend that retinal examination starts in infancy but there are practical issues with retinal imaging in very young children and a compromise would be to start retinal surveillance at 2–4 years of age [2, 6]. The risk of early onset phaeochromocytoma/paraganglioma could be mitigated by starting surveillance earlier in those at higher risk because of family history or the presence of a Type 2 *VHL* variant (as was happening in some centres) [3, 32, 33].

Though the prevalence of ELST in a carefully screened cohort of individuals with VHL disease was estimated previously at 11% [34], the frequency of ELST in our cohort was only 1.3%. This lower prevalence may reflect, that in 12 centres investigations for ELST were only initiated when patients were symptomatic.

There were 33 deaths over the study period (mean 50.4 years, age range 25–82) and 17 deaths were known to have been related to VHL disease (mean age at death 48.3 years, range 25–68 years), Our methodology did not allow us to estimate life-expectancy but we note that at least 32% of deaths were unrelated to VHL disease and, a paper from Denmark studying deaths from VHL disease between 1841 and 2010 found that 79% deaths were secondary to VHL disease but the risk of a VHL-related death decreased over time [35].

There are a number of limitations to this audit. Information was gathered through clinical genetics services and so ascertainment was likely incomplete (patients not referred for genetic testing and management would not have been ascertained). Previous prevalence estimates of VHL disease are at 1 in 53,000 in the East of England, 1 in 91,111 in north-west England, ~1 in 39,000 in the district of Freiburg in Germany and ~1 in 47,000 in Denmark [5, 10, 36, 37]. The maximum prevalence from our study was estimated ~1 in 69,000 which is intermediate with the two previous UK estimates (the high prevalence in the district of Freiburg presumably reflects the frequency of the “Black Forest (p.Tyr98His founder mutation in the region) [38]. This suggests that our ascertainment at a national level was in line with that from previous local studies. Though information was collected on a standard proforma it was completed by a different individual(s) in each centre and so we cannot exclude some variability in how information was collected and interpreted. The study was performed as a clinical audit and so there were restrictions on the availability of individual level data (e.g. information on *VHL* variants was not recorded). Information on the frequency and causes of patient factors non-compliance with screening protocols was not collected and would be an important issue to address in future investigations. Due to the cross-sectional nature of the study, some of the patients had only had a single round of surveillance. Despite these limitations, this study provides novel information that complements that produced from research cohorts for which there are likely to be significant ascertainment bias and it provides a profile of routine clinical care for a rare cancer predisposition syndrome at a national level in a country with a universal healthcare system. Although the UK does not have a centrally-commissioned specification for VHL surveillance clinics, there was considerable similarity between centres in the surveillance they offered. One outcome of this audit may be to reduce variability between centres. With the advent of HIF-2 antagonists for the medical management of VHL disease, the data from this study will be invaluable for costing and planning new developments in VHL care pathways.

## Supplementary information

Supplementary material

Reproducibility check list

## Data Availability

Data are available to bona fide researchers from the corresponding authors.
