# Supplementary material for: Evaluation of tumour surveillance protocols and outcomes in von Hippel-Lindau disease in a national health service
Source: Br J Cancer. 2022 Feb 19;126(9):1339–45. doi: 10.1038/s41416-022-01724-7 (PMC8857742; doi:10.1038/s41416-022-01724-7)
Supplement: Supplementary file 1 — Supplementary material [file 41416_2022_1724_MOESM1_ESM.docx]

SUPPLEMENTARY MATERIAL

Questionnaire used to collect data

**Clinical Genetics National VHL audit 2018**

**Background**

Following a discussion at the national clinical leads meeting it was agreed that an audit of VHL screening practice in the UK should be carried out to establish current practice and determine whether there are significant regional differences and the potential impact, if any, these differences may have on measurable clinical outcomes.

The audit is designed to record current VHL screening practice with limited information being gathered about clinical outcomes. Regional genetics centres currently undertake most VHL surveillance. In a few centres, VHL screening is carried out by other specialties which will also be asked to take part.

Patient identifiable data will not be collected.

Information about certain complication rates will be gathered to determine whether there is significant variation that may be related to screening recommendations and practice e.g. abdominal MRI for pancreatic neuroendocrine tumours.

The audit has been developed by Richard Sandford, Eamonn Maher and Fiona Lalloo, approved by Cambridge University Hospitals NHS Foundation Trust and reviewed by VHL UK/Ireland.

**Eligibility**

- Individuals of any age with a clinical and/or molecular diagnosis of VHL disease OR
- Individuals of any age at 50% risk of inheriting VHL who have not undergone predictive genetic testing AND
- who have been under follow up for all or part of the period 2012-2017.

**FAQs:**

- A clinical diagnosis of VHL is made based on the following criteria described in Genereviews (<https://www.ncbi.nlm.nih.gov/books/NBK1463/)>:

1. A simplex **case** (i.e., an individual with no known family history of VHL syndrome) presenting with **two or more** characteristic lesions:

two or more haemangioblastomas of the retina, spine, or brain

or

a single haemangioblastoma in association with a visceral manifestation (e.g., renal cell carcinoma, adrenal or extra-adrenal phaeochromocytoma, endolymphatic sac tumour (ELST), or neuroendocrine tumour of the pancreas (pNET)

1. An individual with a positive family history of VHL syndrome in whom one or more of the following syndrome manifestations is present:

- Retinal angioma
- Spinal or cerebellar haemangioblastoma
- Adrenal or extra-adrenal pheochromocytoma
- Renal cell carcinoma
- Multiple renal and pancreatic cysts

The audit will be carried out against screening guidelines published in Maher *et al.* Eur J Hum Genet. 2011 Jun; 19(6): 617–623.

| *Screen for retinal angioma:* Annual ophthalmic examinations (direct and indirect ophthalmoscopy), beginning in infancy or early childhood. |
| --- |
| *Screen for CNS haemangioblastoma:* MRI scans of the head for every 12–36 months, beginning in adolescence. |
| *Screen for renal cell carcinoma and pancreatic tumours:* MRI (or ultrasound) examinations of the abdomen every 12 months, beginning from the age of 16 years. |
| *Screen for phaeochromocytoma:* Annual blood pressure monitoring and 24-h urine studies for catecholamine metabolites. More intense surveillance (e.g. annual measurement of plasma normetanephrine levels, adrenal imaging, beginning from the age of 8 years should be considered in families at high-risk for phaeochromocytoma). |

(Additional investigations may be instigated in response to symptoms or signs of specific complications (e.g., ELSTs)).

- For individuals at 50% risk, record screening offered before predictive genetic test carried out. These individuals can also be entered if a positive predictive test is carried out.
- Please record all patients attending for all or part of the time period
- When asked for age at commencement of screening please enter age or a range if appropriate e.g. 16-18 years but please avoid terms such as infancy or adolescence.
- When describing the frequency of a screening test please describe either in terms of frequency in months/years or using other terms such as ‘if symptomatic’ or ‘at patient request’ or ‘at baseline only’.
- If a patient has multiple lesions please use a separate row for each one if possible or record ‘multiple’ if it is not possible to record data on each lesion.

**National VHL screening audit 2018 Proforma**

**Eligibility**

- Individuals with a molecular or clinical diagnosis of VHL OR
- Individuals at 50% risk of inheriting VHL who have not undergone predictive genetic testing AND
- Individuals who have been under follow up for all or part of the period 2012-2017.

**Centre: xxxxxxx**

**Contact details of audit lead:**

- **Name xxxxxx**
- **Email xxxxxx**
- **Phone xxxxx**

(Please add rows to tables as required)

1. Number of patients with a clinical or molecular diagnosis of VHL seen in your clinical service during 2012-2017?

| Total number |  |
| --- | --- |
| Male |  |
| Female |  |
| Age range |  |
| Number with a pathogenic VHL mutation |  |
| Number who are mutation negative |  |

1. Number of individuals at 50% risk of inheriting VHL who have not had predictive genetic testing after their first screening visit or where a familial mutation has not been identified who attend for screening?

| Total number |  |
| --- | --- |
| Male |  |
| Female |  |
| Age range |  |
| Number with known familial VHL mutation |  |

1. Which clinical specialities attend the VHL screening clinic? Please identify the lead speciality. Tick all that apply

| Specialty | Attend clinic | Lead specialty  (tick one) | Consultant | Other e.g. genetic counsellor |
| --- | --- | --- | --- | --- |
| Genetics |  |  |  |  |
| Ophthalmology |  |  |  |  |
| Endocrinology |  |  |  |  |
| Neurosurgery |  |  |  |  |
| ENT |  |  |  |  |
| Urology |  |  |  |  |
| Paediatrics |  |  |  |  |
| Other |  |  |  |  |

1. Please describe your screening protocol^a^:

|  | Method^1^ | From age^2^ | Frequency^3^ |
| --- | --- | --- | --- |
| Eyes |  |  |  |
| Phaeochromocytoma |  |  |  |
| Renal cell carcinoma |  |  |  |
| Pancreatic neuroendocrine tumour |  |  |  |
| Cerebellar Haemangioblastoma |  |  |  |
| Spinal Haemangioblastoma |  |  |  |
| Endolymphatic sac tumour |  |  |  |
| Other: |  |  |  |
| Blood pressure |  |  |  |
|  |  |  |  |
|  |  |  |  |
|  |  |  |  |

^a^only for screening and not for follow up or investigation of lesions identified by screening

^1^e.g. ultrasound and/or MRI, CT. **If this has changed please add details and date of change**.

^2^ or provide a range in years only

^3^ e.g. annual, 2 yearly, or if symptomatic

1. Please list the investigations that are carried out ‘on the day’ of the screening appointment

| Investigation | Tick all those that apply |
| --- | --- |
| Retinal examination |  |
| Plasma metanephrines |  |
| Urinary metanephrines  (bottles given or collected) |  |
| Renal Ultrasound |  |
| Other (please list) |  |
|  |  |
|  |  |

1. Do you measure blood pressure at each clinic visit? If so from what age?

| From age |  |
| --- | --- |
| Method: |  |
| Manual |  |
| Automated |  |
| Resting/sitting |  |
| Sitting and standing |  |

1. Do you offer screening for any other complications?

| Complication | Screening investigation |
| --- | --- |
|  |  |
|  |  |
|  |  |
|  |  |
|  |  |
|  |  |

1. How many VHL patients have died during this period? Please provide the cause of death if known and whether this was directly related to their VHL.

| Patient (local ID) | Cause of death | Age at death | VHL related Y/N |
| --- | --- | --- | --- |
|  |  |  |  |
|  |  |  |  |
|  |  |  |  |
|  |  |  |  |
|  |  |  |  |

**The following questions relate to screening for CNS haemangioblastomas and ELSTs during 2012-2017:**

1. How many patients with a cerebellar or spinal haemangioblastoma required surgery during this period (2012-2017)? Please give age at surgery for each lesion and state whether initially symptomatic or detected via screening?

| **Patient (local ID)** | **Cerebellar** | **Spinal** | **Age** | **Symptomatic or screening?** |
| --- | --- | --- | --- | --- |
|  |  |  |  |  |
|  |  |  |  |  |
|  |  |  |  |  |
|  |  |  |  |  |
|  |  |  |  |  |
|  |  |  |  |  |
|  |  |  |  |  |
|  |  |  |  |  |
|  |  |  |  |  |
|  |  |  |  |  |
|  |  |  |  |  |
|  |  |  |  |  |
|  |  |  |  |  |
|  |  |  |  |  |
|  |  |  |  |  |
|  |  |  |  |  |
|  |  |  |  |  |
|  |  |  |  |  |
|  |  |  |  |  |
|  |  |  |  |  |
|  |  |  |  |  |
|  |  |  |  |  |

1. How many patients were diagnosed with an ELST during this period?

| Number | Symptomatic | Screening |
| --- | --- | --- |
|  |  |  |

**The following questions relate to screening for renal cell carcinoma (RCC) from 2012-2017:**

1. In this time period, 2012-2017, how many patients with an RCC or multiple RCCs have been identified? Please specify for each patient the age, initial tumour diameter if documented and imaging modality used that detected the RCC.

| Patient (local ID only) | Diameter | Imaging modality | First CT or MRI Y/N | Treatment modality (including observation) | Age at presentation | Age at removal (if applicable) |
| --- | --- | --- | --- | --- | --- | --- |
|  |  |  |  |  |  |  |
|  |  |  |  |  |  |  |
|  |  |  |  |  |  |  |
|  |  |  |  |  |  |  |
|  |  |  |  |  |  |  |
|  |  |  |  |  |  |  |
|  |  |  |  |  |  |  |
|  |  |  |  |  |  |  |
|  |  |  |  |  |  |  |
|  |  |  |  |  |  |  |
|  |  |  |  |  |  |  |
|  |  |  |  |  |  |  |
|  |  |  |  |  |  |  |
|  |  |  |  |  |  |  |
|  |  |  |  |  |  |  |
|  |  |  |  |  |  |  |
|  |  |  |  |  |  |  |
|  |  |  |  |  |  |  |

1. At what stage (tumour size) do you refer a potential RCC to urology for further evaluation?

|  |
| --- |

1. How many patients have developed metastatic RCC during the course of their screening in this period? For each patient please try and identify the size of the tumour at initial presentation and the initial treatment offered.

| Patient (local ID only) | Age at diagnosis of metastatic disease | Age at initial diagnosis of RCC (if different) | Initial treatment | Initial size at diagnosis | Metastatic disease sites |
| --- | --- | --- | --- | --- | --- |
|  |  |  |  |  |  |
|  |  |  |  |  |  |
|  |  |  |  |  |  |
|  |  |  |  |  |  |
|  |  |  |  |  |  |

1. How many patients have developed end stage renal disease and received dialysis or transplantation as a direct result of their VHL disease? Please specify the age at which this occurred?

| Patient (local ID only) | Age at ESRD | First treatment modality (HD or Transplant) | Age at Transplantation |
| --- | --- | --- | --- |
|  |  |  |  |
|  |  |  |  |
|  |  |  |  |
|  |  |  |  |
|  |  |  |  |

**The following questions relate to screening for pancreatic neuroendocrine tumours from 2012-2017:**

1. How many patients with a pancreatic neuroendocrine tumour(s) have been identified? Please specify for each patient, the imaging modality used that detected the pNET. If CT or MRI, was this the first time abdominal CT or MRI had been offered?

| Patient (local ID only) | Diameter | Imaging modality | First MRI or CT scan Y/N | Age at diagnosis | Observation or Treatment modality including pancreatectomy | Age at pancreatectomy |
| --- | --- | --- | --- | --- | --- | --- |
|  |  |  |  |  |  |  |
|  |  |  |  |  |  |  |
|  |  |  |  |  |  |  |
|  |  |  |  |  |  |  |
|  |  |  |  |  |  |  |
|  |  |  |  |  |  |  |
|  |  |  |  |  |  |  |
|  |  |  |  |  |  |  |
|  |  |  |  |  |  |  |

1. How many patients presented with or developed metastatic disease?

| Patient (local ID only) | Metastatic disease sites | Initial size at diagnosis | Initial treatment or surveillance | Age at first diagnosis | Age at diagnosis of metastatic disease |
| --- | --- | --- | --- | --- | --- |
|  |  |  |  |  |  |
|  |  |  |  |  |  |
|  |  |  |  |  |  |
|  |  |  |  |  |  |
|  |  |  |  |  |  |

**For patients at 50% risk of inheriting VHL with no clinical features:**

1. Are your screening criteria the same as those above?

Yes

1. If not, how do they differ?

-

1. When do you stop screening?

**Does your service routinely see patients with an apparently isolated CNS haemangioblastoma?**

**Yes If yes, are there any age criteria?**

**For patients with an isolated CNS haemangioblastoma and a negative VHL gene test during 2012-2017:**

1. How many have been screened?
2. Are your screening criteria the same as those above?
3. If not, how do they differ?
4. Do you offer screening to other family members and if so, who?
5. At what age do you discontinue screening if no other features of VHL are found?

**Supplementary questions:**

**Earliest age of diagnosis of VHL complications**

1. Have any of your patients had a retinal angioma before 5 years of age? If so please specify age at diagnosis and whether the angioma was detected because of symptoms or detected by screening.

| Number diagnosed < 5 years | Number identified by screening | Number presenting with symptoms |
| --- | --- | --- |
|  |  |  |

1. Have any of your patients had a CNS haemangioblastoma before 5 years of age? If so please specify age at diagnosis and whether the lesion was detected because of symptoms or detected by screening.

| Number diagnosed < 5 years | Number identified by screening | Number presenting with symptoms |
| --- | --- | --- |
|  |  |  |

1. Have any of your patients had a renal tumour before 18 years of age? If so please specify age at diagnosis and whether the lesion was detected because of symptoms or detected by screening.

| Number diagnosed < 18 years | Number identified by screening | Number presenting with symptoms |
| --- | --- | --- |
|  |  |  |

1. Have any of your patients had a phaeochromocytoma/paraganglioma before 10 years of age? If so please specify age at diagnosis and whether the lesion was detected because of symptoms or detected by screening.

| Number diagnosed < 10 years | Number identified by screening | Number presenting with symptoms |
| --- | --- | --- |
|  |  |  |

1. What was the earliest age of diagnosis of a pNET, either single of multiple? Was it detected because of symptoms or detected by screening.

| Age at diagnosis | Identified by screening | Presenting with symptoms |
| --- | --- | --- |
|  |  |  |
